# A Rare Case of a Non-strangulated Bladder Pantaloon Hernia

**DOI:** 10.7759/cureus.31208

**Published:** 2022-11-07

**Authors:** Innocent Lutaya, Anam Sayed Mushir Ali, Cristina Terron, Jonatan Habart, Guilherme Torres, Raul Mederos

**Affiliations:** 1 Medical School, American University of Antigua, New York City, USA; 2 Medical School, Indian Institute of Medical Science and Research, Aurangabad, IND; 3 Medical School, Florida International University/Hialeah Hospital, Hialeah, USA; 4 Surgery, Hialeah Hospital, Hialeah, USA

**Keywords:** romberg, saddlebag, mesh repair, strangulated, bladder, bladder hernia, indirect hernia, hernia, pantaloon, pantaloon hernia

## Abstract

A pantaloon hernia occurs when an indirect and direct hernia develop at the same time. The urinary bladder is a rare component of pantaloon hernias. There is a lack of literature regarding an ipsilateral pantaloon with a herniated urinary bladder. Clinically, it has a vague presentation associated with abdominal pain and urinary retention symptoms. The best diagnostic modality is an abdominal CT scan. Surgery is the treatment of choice, rendering a good prognosis. Untreated bladder hernia may lead to strangulation and necrosis of the urinary bladder. We present a rare case of a right-sided pantaloon hernia with a bladder herniation in a 65-year-old man.

## Introduction

A pantaloon hernia is defined as a combination of two adjacent hernia sacs in the inguinal and or femoral regions on the same side. The sacs are thus present on both sides of the inferior epigastric vessels [1]. Almost 75% of abdominal wall hernias are inguinal hernias; indirect being the most common hernia in both genders and overall. The incidence of pantaloon hernia in males is 5.6%, and in females, it is approximately 1.8%. A diagnosis of urinary bladder herniation is reported in about 1-4% of all inguinal hernia cases [2]. Urinary bladder herniation may also occur in an acquired direct inguinal hernia where the bladder is pulled into the herniation sac, and accompanied by a sheath of the peritoneum. When compared with the majority of cases which are diagnosed intraoperatively, only 7% of bladder hernias are identified prior to surgery [3]. Diagnosis of hernias includes thorough patient history, physical examination, ultrasound, and a CT scan. Surgical repair is the definitive treatment for symptomatic pantaloon hernias. We present a herniated urinary bladder as a direct component of the pantaloon hernia. 

## Case presentation

A 65-year-old male presented for elective surgery with a four-week history of painful scrotal swelling associated with right lower quadrant abdominal pain. He complained of an associated mild suprapubic tenderness that extended to the scrotal mass. He had a history of urinary symptoms including haematuria and voiding problems. A physical exam of the abdomen showed a right inguinal hernia with a reducible scrotal mass. The patient’s vital signs showed heart rate of 66 bpm, respiratory rate of 20 breaths per minute, axillary temperature of 97.9 degrees Fahrenheit, and blood pressure of 155/88 mmHg.

**Figure 1:**
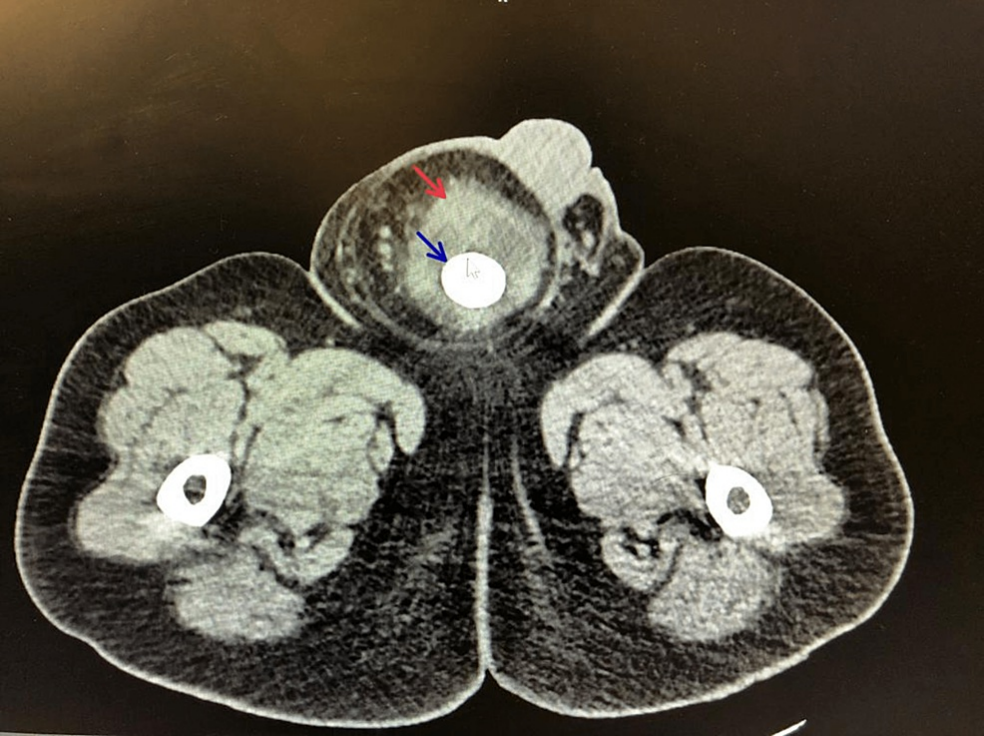
A axial CT scan showing a pantaloon urinary bladder herniation (red arrow) with a calculus (blue arrow).

**Figure 2:**
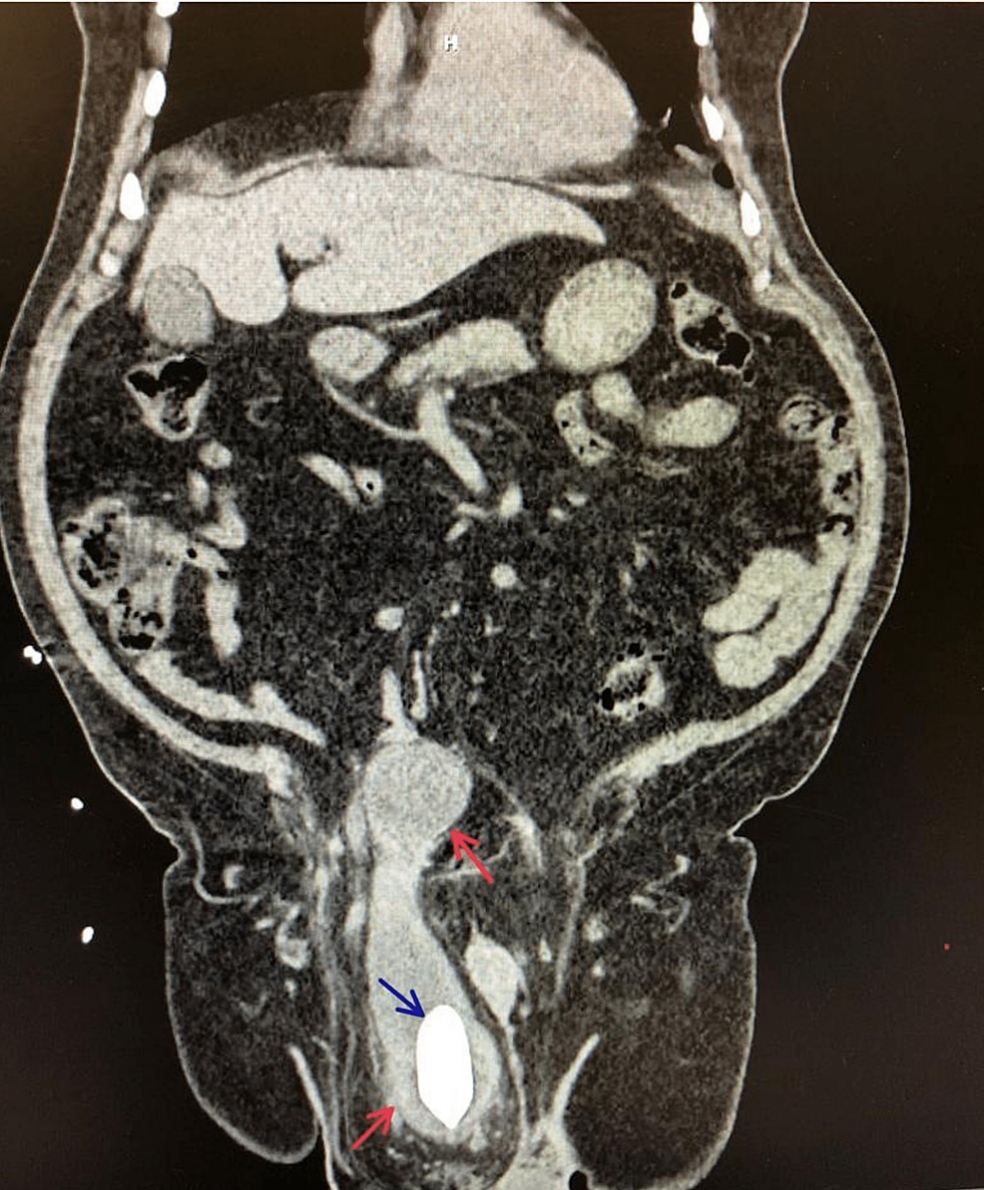
A coronal CT scan showing a pantaloon urinary bladder herniation (red arrows) with a calculus (blue arrow).

**Figure 3:**
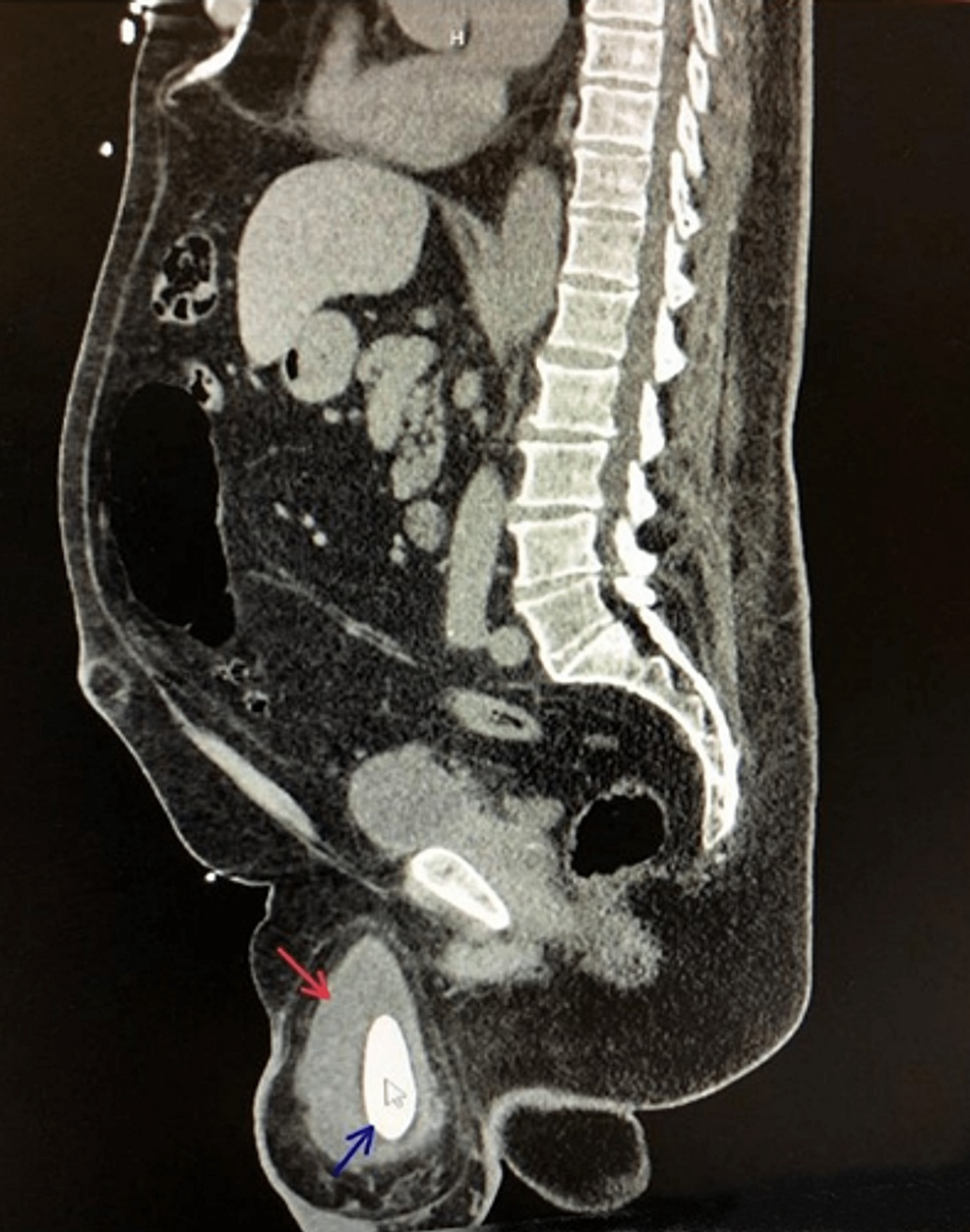
A sagittal CT scan showing a pantaloon urinary bladder herniation (red arrow) with a calculus (blue arrow).

A decision was made to approach this case with imaging studies. A non-contrast abdominal and pelvic computed tomography (CT) scan showed a large right inguinal hernia protruding to the scrotum (Figures 1, 2, 3; red arrows), containing a significant portion of the urinary bladder. A 3 cm calculus (Figures 1, 2, 3; blue arrows), causing an obstructive pattern within the bladder, was detected. Both ureters were dilated distally with mild bilateral hydronephrosis. Upon consultation with the urologist, a consensus was reached to repair the inguinal hernia using a tension-free mesh first and tending to the urinary calculus at a later time.

The patient was admitted to surgery under general anesthesia. A Lichtenstein technique was indicated to repair the hernia by applying a polypropylene mesh to decrease the risk of recurrence. The patient tolerated the procedure well without any complications and the follow-up was uneventful. A decision was made not to open the bladder to remove the calculus during this surgery in order to minimize the risk of bacterial contamination of the mesh and surgical field. The patient was sent home two days postoperatively, and was also referred to urology for a further evaluation, cystoscopy, and stone removal after recovery. 

## Discussion

A pantaloon hernia or a saddlebag hernia is characterized by a combination of both indirect and direct hernias ipsilaterally. A pantaloon hernia has an incidence of three times more in males than females [2]. This case included a herniated urinary bladder as a direct component of the pantaloon hernia. 

A herniated bladder occurs in a direct inguinal hernia, wherein the sac contents include the bladder along with a layer of peritoneum. It is implicated in around 2-4% of diagnosed cases of inguinal hernia and its incidence increases up to 10% in the elderly male population [4,5]. The most common pathophysiological risk factors include obesity, decreased tone of the bladder, and chronic obstruction. Other comorbidities which may contribute to the condition include benign prostatic hyperplasia (BPH) or chronic obstructive pulmonary disease (COPD). The occurrence of a bladder hernia is usually associated with prominent urinary symptoms, which show improvement after the repair of the hernia. The major associated comorbidities include bilateral hydronephrosis and stone within the herniated bladder, as seen in our patient. Further complications include renal failure, vesicoureteral reflux, bladder rupture, strangulation, necrosis, and abscess formation. The rate of these serious complications rises up to 24% of cases [6]. The diagnosis of this type of hernia has proven to be very difficult. The majority of them are diagnosed perioperatively, 16% postoperatively, while less than 7% of them are diagnosed preoperatively [7]. Discovery is usually incidental, often during a hernia repair, and has an estimated 12% risk of injury [8]. An early diagnosis can help prevent multiple surgical risks and complications. In a study conducted in 2004, 23% of the 190 cases diagnosed with herniated bladder were associated with various complications, while 11.2% were reported to be associated with urological malignancies [6]. Therefore early diagnosis via radiological imaging plays an important role in the prevention of these complications [8,9]. 

Radiological investigation is not a usual diagnostic modality for inguinal hernias, more so for the detection of a bladder hernia. Cystography is the gold standard for diagnosis [10,11]. After a diagnosis, a CT scan can be carried out for further surgical planning. The standard modality of treatment is a herniorrhaphy, wherein the bladder is surgically reduced [10,9]. After identification and proper dissection, it is followed by the repair of the direct component of the hernia according to the preference of the surgeon. Bladder resection can be done in cases complicated by necrosis of the bladder wall, a diverticulum, or the occurrence of a tumor in the herniated section of the bladder [10,12,13]. In certain cases where the patient may choose conservative treatment, if no prominent urinary symptoms are experienced, intermittent urethral catheterization may also be carried out.

## Conclusions

Pantaloon bladder hernias are a rare occurrence that have vague clinical presentations. Patients usually have localized mild abdominal pain, along with urinary symptoms such as recurrent urinary tract infections, and bowel obstruction due to herniation. Complications can be grave such as incarceration, strangulation, and necrosis. It is therefore important to consider a bladder hernia as part of differential diagnoses and offer prompt treatment given this constellation of symptoms to reduce the risk of necrosis, sepsis, and death.
